# The effect of short-term training about depth predicting score on the diagnostic ability of invasion depth for differentiated early gastric Cancer among non-expert endoscopists

**DOI:** 10.1186/s12909-023-04230-3

**Published:** 2023-05-17

**Authors:** Hui Li, Hui Hu, Ping Geng, Panhui Guo, Yuanrong Zhu, Lulu Zeng, Jun Liu, Xiangpeng Hu

**Affiliations:** 1 Digestive Department, the Second Affiliated Hospital of Anhui Medical University, Anhui Medical University, Hefei, Anhui Province 230601 China; 2 Department of Pathophysiology, School of Basic Medical College, Anhui Medical University, Hefei, Anhui Province 230032 China; 3 Functional experiment center, School of Basic Medical College, Anhui Medical University, Hefei, Anhui Province 230032 China

**Keywords:** Early gastric cancer, Endoscopists, Training, Invasion depth, Depth-predicting score

## Abstract

**Background:**

The depth-predicting score (DPS) was proposed based on conventional white-light imaging (C-WLI) endoscopic features of early gastric cancer (EGC) to determine the invasion depth of the neoplasm. However, the effect of DPS on training endoscopists remains unclear. Therefore, we aimed to investigate the effect of short-term DPS training on improving the diagnostic ability of EGC invasion depth and compare the training effect among non-expert endoscopists at different levels.

**Methods:**

In the training session, the definitions and scoring rules of DPS were instructed, and classic C-WLI endoscopic example graphics were exhibited to the participants. Another C-WLI endoscopic images of 88 cases of histologically proven differentiated EGC were selected as an independent test dataset for evaluating the training effect. Each participant was tested, and the diagnostic accuracy rate of invasion depth was calculated differently one week before the training and after the completion of training.

**Results:**

A total of 16 participants were enrolled and completed the training. Participants were divided into a trainee group and a junior endoscopist group according to the total number of C-WLI endoscopies performed. The total number of C-WLI endoscopies performed showed a significant difference between the trainee group and junior endoscopist group (350 vs. 2500, *P* = 0.001). No significant difference between the trainee group and junior endoscopist group was observed for pre-training accuracy. The overall diagnostic accuracy of invasion depth was improved significantly after completing DPS training compared with before (68.75 ± 5.71% vs. 61.58 ± 9.61%, *P* = 0.009). In the subgroup analysis, the post-training accuracy was higher than the pre-training accuracy, but significant improvement was observed only in the trainee group (61.65 ± 7.33% vs. 68.32 ± 5.71%, *P* = 0.034). In addition, no significant difference in post-training accuracy between the two groups was observed.

**Conclusion:**

Short-term DPS training can improve the diagnostic ability of the invasion depth of EGC and homogenize the diagnostic ability of non-expert endoscopists at different levels. The depth-predicting score was convenient and effective for endoscopist training.

## Introduction

Gastric carcinoma (GC) is one of the foremost prevalent malignant neoplasms of the digestive tract and is the fourth leading cause of cancer-related deaths worldwide [1–3]. Early gastric cancer (EGC) is defined as invasive gastric adenocarcinoma confined to the mucosa or submucosa, irrespective of lymph node metastasis (T1, any N) [4]. The prognosis of EGC is excellent for patients, with a 5-year survival rate following lesion resection is > 90% [5, 6]. Minimally invasive treatments, including endoscopic submucosal dissection (ESD), have been widely accepted as the first-line treatment modality for EGC because they are less invasive and offer a better quality of life compared to surgical excision [7, 8].

The selection of curative endoscopic resection for EGC depends on the differentiation, size, and depth of invasion of the lesions [9, 10]. The absolute indications for curative endoscopic resection are differentiated EGC less than 20 mm in diameter and confined to the mucosa, with no lymphatic or vascular involvement [10]. Therefore, accurate determination of the depth of tumor invasion is a crucial indicator for selecting the optimal therapeutic strategy for EGC patients [11–13]. Currently, conventional white-light imaging (C-WLI), magnifying endoscopy (ME), and endoscopic ultrasonography (EUS) are the main endoscopic imaging modalities used to predict the invasion depth of EGC [14].

Because of its accessibility and convenience, C-WLI endoscopy is more commonly used to predict the invasion depth of EGC in clinical practice [15]. The depth-predicting score (DPS), which is based on four main endoscopic features, has been proposed for use in diagnosing the invasion depth of differentiated EGC during C-WLI endoscopy. The four features include remarkable redness, uneven surface, lesion size more than 30 mm and margin elevation. It has been reported that the diagnostic accuracy of DPS achieved over 80% and DPS has the superiority of convenience, simplicity, low cost and no additional equipment required, which makes it widely used for invasion depth diagnosis [16]. However, the effect of DPS used for endoscopist training is unclear. Thus, we designed this study to evaluate the effect of short-term DPS training on improving the diagnostic ability of the EGC invasion depth and compare the training effect among non-expert endoscopists at different levels.

## Methods

### Participants

Eligible participants were gastrointestinal endoscopists from the Second Affiliated Hospital of Anhui Medical University who had not accepted a professional training course about EGC diagnosis and did not know DPS before this study. Participants gave their informed consent prior to starting the survey. All participants were asked to complete a survey questionnaire including age, gender, years of experience in C-WLI endoscopy, and the number of C-WLI endoscopies performed. Exclusion criteria were incomplete questionnaires, unwillingness to participate, and had accepted a professional training course about EGC diagnosis before this study.

### Training program and assessment

The training program and assessment consisted of three steps. Stage 1 (pre-training test): All participants were asked to take the diagnostic test by the test dataset first before the training. Stage 2(short-term training): One week after pre-training test, the definitions and scoring rules of DPS were introduced and explained, and classic C-WLI endoscopic example graphics were exhibited to the participants. Stage 3 (post-training test): All participants took the second diagnostic test by the same test dataset immediately after training. The diagnostic accuracy rate of each participant was calculated separately before the training and after the completion of training as evaluation metrics. Using the histological invasion depth as gold standard, the diagnostic accuracy was obtained by dividing the number of cases judged correctly before or after training by the total number of cases in the test dataset. Because SM2 patients can be excluded from absolute indication of ESD, specificity to predict M-SM1 may be more important in this situation. The specificity to predict M-SM1 was also calculated separately before and after the training. Using the histological invasion depth as gold standard, the specificity was obtained by dividing the number of M-SM1 EGC cases judged correctly before or after training by the actual number of M-SM1 EGC cases.

The dataset of the diagnostic test comprised C-WLI endoscopic images of 88 cases of histologically proven differentiated EGC with complete medical record materials. Participants were blinded to the clinical and pathological information. We used the same 88 cases in the same order for the pre-training test and post-training test, and this information was not disclosed to participants. All lesion images were collected with close-up and distant views. All endoscopic images were taken by C-WLI endoscopy (video-endoscope GIF-H260, GIF-H290; Olympus Medical Systems, Tokyo, Japan or video-endoscope EG29-i10N, Pentax, Tokyo, Japan) during actual clinical practice.

### Details of DPS

DPS includes four endoscopic metrics: remarkable redness, uneven surface, lesion size more than 30 mm and margin elevation. Each endoscopic metric was assigned a certain number of points. Remarkable redness or uneven surface was assigned 1 point, and lesion size more than 30 mm or margin elevation was assigned 2 points. The resulting DPS was measured by a 6-point scale. A total of 3 points was defined as the cut-off value between pathologic M-SM1 and SM2. Written definitions and assigned points of each endoscopic feature were made available to all participants during DPS training and the post-training test (Table 1).

**Table 1:** Description of Endoscopic Features used in the DPS

Written Definitions and Assigned Points of Each Endoscopic Feature		
Remarkable redness	A reddish area similar to regenerative epithelium	1 point
Uneven surface	Nodulations in the tumor’s surface	1 point
Margin elevation	A protruding edge surrounding the tumors	2 points
Tumor size > 30 mm	Lesion size more than 30 mm	2 points

### Statistical analysis

Statistical data analysis was performed using SPSS, version 23.0 (SPSS Inc.). The Shapiro–Wilk test was used to detect the normality of variables. Data were expressed as percentages or mean ± standard deviation, medians and interquartile ranges (1st quartile, 3rd quartile) as appropriate. Fisher’s exact test and Wilcoxon rank-sum tests were used to compare inter-group differences in demographic data. For normally distributed data, inter-group differences were compared using t-test for independent samples. Paired t tests were used to compare pre- and post-training differences. All statistical tests were two-sided, and P < 0.05 was considered statistically significant.

## Results

A total of 16 participants were enrolled and completed the training. Participants were divided into a trainee group and a junior endoscopist group according to the total number of C-WLI endoscopies performed. Trainees were defined as residents-in-training who had completed < 400 previous C-WLI endoscopies. Junior endoscopists were defined as those who had completed 2000 to 3000 C-WLI endoscopies. The 16 learners included 8 trainees and 8 junior endoscopists. Participants in the trainee group were on average 26 years old (range, 23–27), the majority were female (75%), and they had less than 1 year of operator experience. Participants in the junior endoscopist group were on average 33 years old (range, 30–36), all were female (100%), and 37.5% had more than 3 years of operator experience. The total number of C-WLI endoscopies performed in the career was significantly different between the trainee group and the junior endoscopist group (350 vs. 2500, respectively, P = 0.001). The demographic information and characteristics are summarized in Table 2.

**Table 2:** Demographics and Operational Experience of Participants

Characteristic	Junior Endoscopists (n = 8)	Trainees (n = 8)	*P*-value
**Age(y)**	32.5(32-34.75)	27(24.5–27)	0.001
**Gender**			0.467
Male	0(0)	2(25)	
Female	8(100)	6(75)	
**Operating year**			
Less than 1	NA	8(100)	
2	5(62.5)	NA	
More than 3	3(37.5)	NA	
**TWLE performed in career**	2500(2,000–3,000)	350(320–350)	0.001

NA, not applicable; TWLE, total white light endoscopy

Data are median (P25, P75) or n (%)

## Pre- and post-training comparison of overall accuracy

**Fig. 1:**
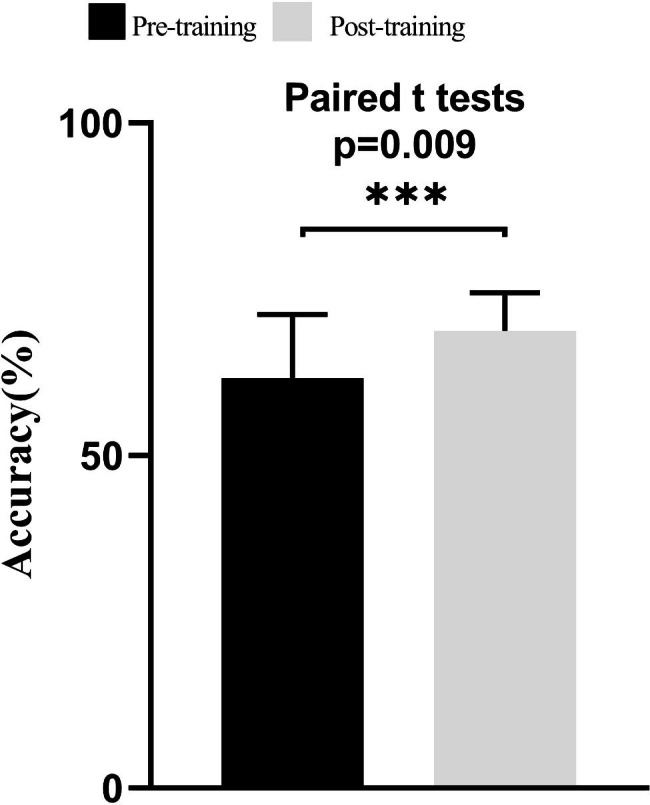
Comparison of overall pre- and post-training diagnostic accuracy. The overall diagnostic accuracy of pre-training and post-training. Diagnostic accuracy was compared using the paired t test. The diagnostic accuracy achieved after training was significantly higher than that of the pre-training test. *p < 0.05

The pre-training test accuracy was described as a baseline level. The overall diagnostic accuracy was significantly higher than the baseline after DPS training (68.75 ± 5.71% vs. 61.58 ± 9.61%, respectively), with a mean difference of 7.17% (95% CI 2.07–12.28, P = 0.009) (Fig. 1).

## Subgroup analysis of pre- and post-training accuracy

**Fig. 2:**
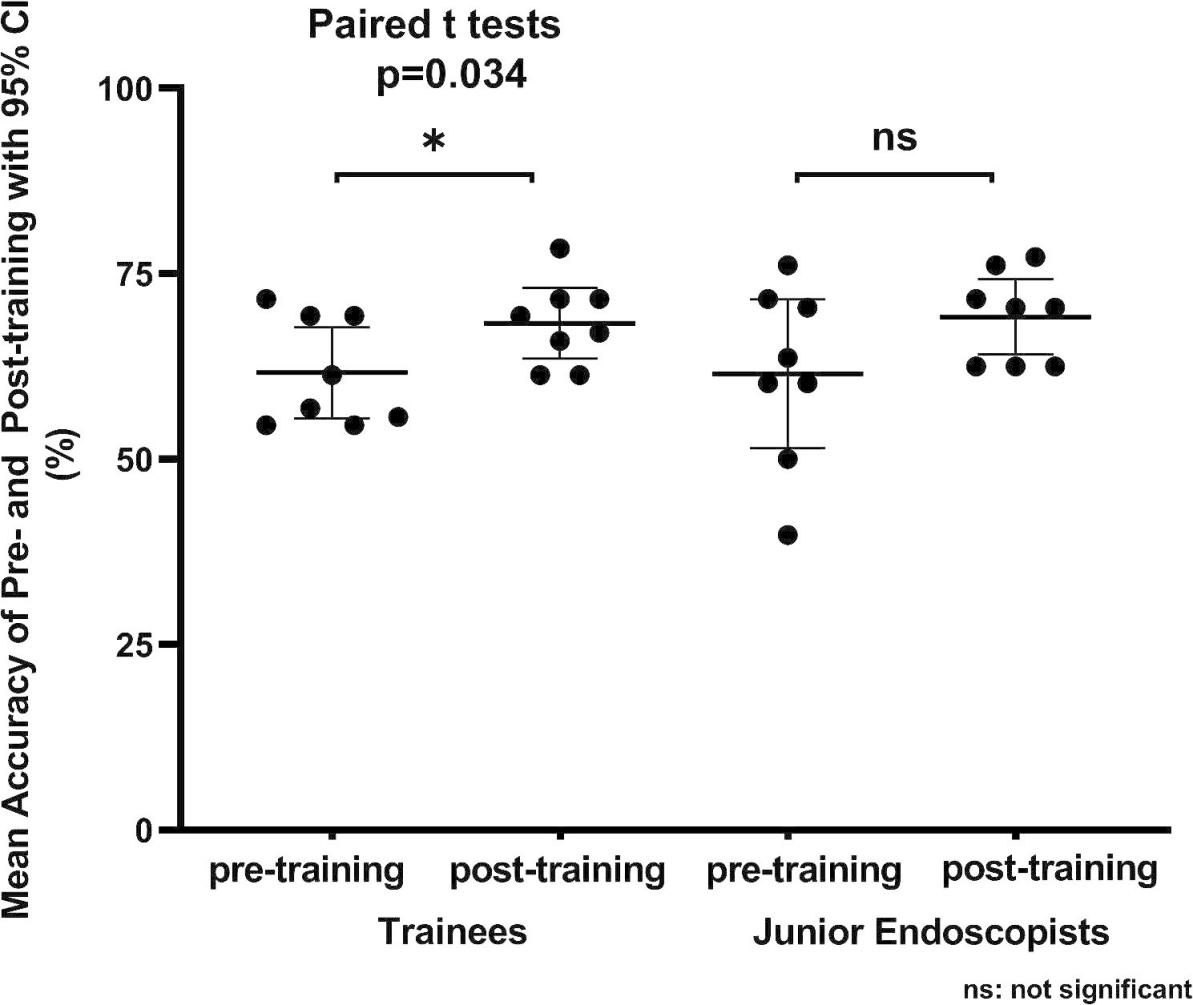
Comparison of pre- and post-training diagnostic accuracy within each group. Mean accuracy of pre-training and post- training (together with 95% confidence intervals) of the trainee group and junior endoscopist group. No significant differences were observed between the two groups before and after training. The accuracy of both groups increased after training, but a statistically significant difference was only found in the trainee group. (ns) not significant, *p < 0.05

In the subgroup analysis, no significant differences between the two groups were observed for pre-training accuracy (61.65 ± 7.33% vs. 61.51 ± 12.0%, P = 0.978) and post-training accuracy (68.32 ± 5.71% vs. 69.18 ± 6.06%, P = 0.776) (Fig. 2). The post-training accuracy was significantly higher than the baseline (68.32 ± 5.71% vs. 61.65 ± 7.33%, P = 0.034) in the trainee group (Fig. 2). In the junior endoscopists’ group, the post-training accuracy was also higher than the baseline, but with no significant differences (69.18 ± 6.06% vs. 61.51 ± 12.0%, P = 0.114) (Fig. 2).

## Pre- and post-training comparison of specificity to Predict M-SM1 EGC

The pre-training specificity to predict M-SM1 EGC was described as a baseline level. The overall specificity was significantly higher than the baseline after DPS training (73.11±10.47% vs. 61.08±14.48%, P = 0.007). In the subgroup analysis, the post-training specificity was also higher than the baseline both in the trainee group (72.54±11.89% vs. 62.12±9.92%) and the junior group (73.68±9.65% vs. 60.04±16.99%), but with no significant differences.

## Discussion

In this study, the value of short-term DPS training for endoscopists to improve the diagnostic ability of the invasion depth of EGC during C-WLI endoscopy was evaluated. We found that the overall diagnostic accuracy improved after training. It is indicated that short-term DPS training can improve endoscopists’ diagnostic ability of EGC invasion depth, which may be helpful to select the correct treatment strategy for EGC. To the best of our knowledge, this is the first report to apply DPS in endoscopist training and evaluate the effect of DPS training on improving the diagnostic accuracy of invasion depth of EGC.

DPS was proposed upon logistic regression analysis of four main endoscopic features summarized by three expert endoscopists, and the accuracy of the DPS was 82.5–84.8% in the validation set [16]. Although the diagnostic accuracy of DPS in our study did not achieve 70%, this may be explained by the fact that the participants were inexperienced endoscopists without having accepted a professional training course about EGC diagnosis.

In our study, participants were further divided into two groups according to the total number of C-WLI endoscopies performed. We aimed to observe the training effect among non-expert endoscopists with different levels.

On the one hand, we found that prior to training, the junior endoscopist group did not show higher accuracy in determining the invasion depth of EGC than the trainee group, although they performed more total C-WLI endoscopies. This suggested that the total number of endoscopies performed without special training was not correlated with the diagnostic ability of the invasion depth of EGC. Additionally, it was reported in previous studies that training plays an important role in improving the detection rate of EGC [17, 18]. Therefore, special training for endoscopists is very important to improve the diagnostic abilities of invasion depth of EGC.

On the other hand, post-training accuracy in the two groups increased after short-term DPS training, and showed no statistical difference between the two groups. In addition, the standard deviation of the post-training accuracy narrowed after the training. This shows that short-term DPS training helped to achieve homogeneity in the diagnostic abilities of invasion depth of EGC among non-expert endoscopists at different levels.

Although the accuracy of both groups increased after training, a statistically significant difference was not found in the junior group. The reason may be that greater inter-individual variation existed among junior endoscopists before training, as evidenced by the larger standard deviation. Studies have shown that the diagnostic accuracy of invasion depth may largely depend on the knowledge and experience of the endoscopist [19, 20]. For example, tumor size is insensitive in terms of depth of invasion, which may be because size observation under C-WLI endoscopy is greatly influenced by the physician’s technique and equipment [21–23]. Therefore, inter-individual variation in pre-training accuracy became greater in the junior endoscopist group because they had performed more endoscopies with different levels of experience.

Finally, we believed that the main contribution of this study is to show that DPS short-term training can be effective, improve swiftly the diagnostic ability of invasion depth for differentiated EGC among non-expert endoscopists, and have the superiority of being low-cost, simple, and convenient.

## Limitations of the study

The present study has several limitations. First, this was a study performed at a single institution. Second, assessments of the endoscopic staging were performed based on recorded still images rather than real-time endoscopic observation. Third, short-term training was designed in our study. Further studies are needed to observe the effect of using a systematic training atlas and long-term DPS training.

## Conclusion

Short-term DPS training can improve the diagnostic ability of the invasion depth of EGC and homogenize the diagnostic ability of non-expert endoscopists at different levels. The depth-predicting score was convenient and effective for endoscopist training.

## Data Availability

All data generated or analyzed during this study are included in this published article, missing datasets used or analyzed are available from the corresponding author on reasonable request.

## Abbreviations

GC: Gastric carcinoma
EGC: early gastric cancer
DPS: Depth-predicting score
C-WLI endoscopy: Conventional white-light imaging endoscopy
EUS: Endoscopic ultrasonography
M: Mucosa
SM: Submucosa
ESD: Endoscopic submucosal dissection

## References

[1] Sung H, Ferlay J, Siegel RL, Laversanne M, Soerjomataram I, Jemal A et al. Global Cancer Statistics 2020: GLOBOCAN Estimates of Incidence and Mortality Worldwide for 36 Cancers in 185 Countries. *CA Cancer J Clin*, 2021. 71(3): p. 209–249.10.3322/caac.21660 10.3322/caac.21660 33538338

[2] Liu Y, Ding W, Yu W, Zhang Y, Ao X, Wang J (2021). Long non-coding RNAs: Biogenesis, functions, and clinical significance in gastric cancer. Mol Ther Oncolytics.

[3] Machlowska J, Baj J, Sitarz M, Maciejewski R, Sitarz R, Gastric Cancer. Epidemiology, risk factors, classification, genomic characteristics and treatment strategies. Int J Mol Sci. 2020;21(11). 10.3390/ijms21114012. 10.3390/ijms21114012 PMC7312039 32512697

[4] Liu Q, Ding L, Qiu X, Meng F (2020). Updated evaluation of endoscopic submucosal dissection versus surgery for early gastric cancer: a systematic review and meta-analysis. Int J Surg.

[5] Liu B, Sun X (2019). miR-25 promotes invasion of human non-small cell lung cancer via CDH1. Bioengineered.

[6] Choi JH, Kim ES, Lee YJ, Cho KB, Park KS, Jang BK (2015). Comparison of quality of life and worry of cancer recurrence between endoscopic and surgical treatment for early gastric cancer. Gastrointest Endosc.

[7] Karimi P, Islami F, Anandasabapathy S, Freedman ND, Kamangar F (2014). Gastric cancer: descriptive epidemiology, risk factors, screening, and prevention. Cancer Epidemiol Biomarkers Prev.

[8] Pyo JH, Lee H, Min BH, Lee JH, Choi MG, Lee JH (2016). Long-term outcome of endoscopic resection vs. surgery for early gastric Cancer: a non-inferiority-matched cohort study. Am J Gastroenterol.

[9] Gotoda T, Yanagisawa A, Sasako M, Ono H, Nakanishi Y, Shimoda T (2000). Incidence of lymph node metastasis from early gastric cancer: estimation with a large number of cases at two large centers. Gastric Cancer.

[10] Nishizawa T, Yahagi N (2018). Long-term outcomes of using endoscopic submucosal dissection to treat early gastric Cancer. Gut Liver.

[11] Zhang X, Yao J, Zhang Y, Huang X, Wang W, Huang H (2022). Updated evaluation of the diagnostic performance of double contrast-enhanced Ultrasonography in the preoperative T staging of gastric Cancer: a Meta-analysis and systematic review. Front Oncol.

[12] Smyth EC, Nilsson M, Grabsch HI, van Grieken NC, Lordick F (2020). Gastric cancer. Lancet.

[13] Tate DJ, Klein A, Sidhu M, Desomer L, Awadie H, Lee EYT (2019). Endoscopic submucosal dissection for suspected early gastric cancer: absolute versus expanded criteria in a large western cohort (with video). Gastrointest Endosc.

[14] Ochiai Y, Kikuchi D, Inoshita N, Hayasaka J, Suzuki Y, Tanaka M et al. Clinicopathological Features of Advanced Gastric Cancers which Were Misjudged and Subjected to Endoscopic Submucosal Dissection. *Gastroenterol Res Pract*, 2020. 2020: p. 6525098.10.1155/2020/6525098 10.1155/2020/6525098 PMC7071798 32190041

[15] Kato M, Uedo N, Nagahama T, Yao K, Doyama H, Tsuji S (2019). Self-study of the non-extension sign in an e-learning program improves diagnostic accuracy of invasion depth of early gastric cancer. Endosc Int Open.

[16] Abe S, Oda I, Shimazu T, Kinjo T, Tada K, Sakamoto T (2011). Depth-predicting score for differentiated early gastric cancer. Gastric Cancer.

[17] Zhang Q, Chen ZY, Chen CD, Liu T, Tang XW, Ren YT (2015). Training in early gastric cancer diagnosis improves the detection rate of early gastric cancer: an observational study in China. Med (Baltim).

[18] Kim SY, Park JM (2022). Quality indicators in esophagogastroduodenoscopy. Clin Endosc.

[19] Haruki S, Kobayashi K, Yokoyama K, Sada M, Koizumi W. Comparison of diagnostic accuracies of various endoscopic examination techniques for evaluating the invasion depth of colorectal tumors. *Gastroenterol Res Pract*, 2012. 2012: p. 621512.10.1155/2012/621512 10.1155/2012/621512 PMC3471431 23091483

[20] Yao Z, Jin T, Mao B, Lu B, Zhang Y, Li S (2022). Construction and Multicenter Diagnostic Verification of Intelligent Recognition System for endoscopic images from early gastric Cancer based on YOLO-V3 Algorithm. Front Oncol.

[21] Sano T, Okuyama Y, Kobori O, Shimizu T, Morioka Y (1990). Early gastric cancer. Endoscopic diagnosis of depth of invasion. Dig Dis Sci.

[22] Sonnenberg A, Giger M, Kern L, Noll C, Study K, Weber KB (1979). How reliable is determination of ulcer size by endoscopy?. Br Med J.

[23] Yao K, Uedo N, Kamada T, Hirasawa T, Nagahama T, Yoshinaga S (2020). Guidelines for endoscopic diagnosis of early gastric cancer. Dig Endosc.

